# Supplementary material for: The COVID-19 Animal Fostering Boom: Ephemera or Chimera?
Source: Animals (Basel). 2022 May 23;12(10):1325. doi: 10.3390/ani12101325 (PMC9137632; doi:10.3390/ani12101325)
Supplement: Supplementary file 1 [file animals-12-01325-s001.zip › animals-1730966-supplementary.pdf]

Supplementary Material: Level of Foster Service, N=611

	% of Responses
One-time foster	5
Occasional/several times a year	48
Seasonal/kitten season, when not working	3
Frequent/have fosters most of the time	44
